# Histone Modifications in Psychiatry: From Canonical Marks to Neurotransmitter-Mediated Epigenetic Signatures

**DOI:** 10.31083/AP45381

**Published:** 2026-05-21

**Authors:** Weidi Wang, Wei Wang, Xuechen Wang, Zhe Liu, Yu Fang, Peijun Ju, Lei Ding, Daihui Peng

**Affiliations:** ^1^Division of Mood Disorders, Shanghai Mental Health Center, Shanghai Jiao Tong University School of Medicine, 200030 Shanghai, China; ^2^School of Life Science, Shanghai Normal University, 200010 Shanghai, China; ^3^Department of Computer Science and Engineering, East China University of Science and Technology, 200010 Shanghai, China

**Keywords:** histone code, depression, schizophrenia, bipolar disorder, epigenesis, genetic

## Abstract

Histone modifications have emerged as critical epigenetic regulators in the development and progression of mental disorders. This review synthesizes recent advances in the field, highlighting both canonical modifications, such as methylation, acetylation, and phosphorylation, as well as novel discoveries that link neurotransmitter signaling to chromatin regulation. In particular, neurotransmitter-mediated histone modifications, including serotonylation, dopaminylation, and histaminylation, represent a compelling new paradigm by which neuronal activity and environmental stimuli can induce lasting changes in gene expression. Aberrant histone modifications have been implicated in the risk, symptomatology, and treatment response of psychiatric conditions such as depression, bipolar disorder, and schizophrenia. Furthermore, therapeutic strategies that target histone-modifying enzymes, most notably histone deacetylase inhibitors, are being actively explored for their potential to restore epigenetic balance and improve clinical outcomes. A deeper understanding of the mechanistic diversity and disease specificity of histone modifications will be crucial for the development of precision epigenetic therapies in psychiatry.

## 1. Introduction

Histone modifications represent a key epigenetic mechanism that dynamically regulate chromatin structure and gene transcription without altering the underlying DNA sequence. These modifications include acetylation, methylation, phosphorylation, ubiquitination, crotonylation, and serotonylation. They occur predominantly on the N-terminal tails of histone proteins and are catalyzed by specific “writer”, “eraser”, and “reader” enzymes [1]. By altering the accessibility of chromatin, histone modifications play essential roles in fundamental biological processes such as embryonic development [2], cell fate determination [3], and tissue-specific gene regulation [4]. In the central nervous system, they are particularly critical for processes like synaptic plasticity [5], memory formation [6], and neurogenesis [7].

With the expansion of epigenetics research in recent years, the role of histone modifications in mental disorders has attracted more attention [8]. As an important epigenetic regulatory mechanism [9], histone modifications play a crucial role in neural development and in the pathogenesis of mental disorders by influencing chromatin structure and gene expression [8]. Due to their central role in regulating gene expression, histone modifications have emerged as highly promising therapeutic targets for the treatment of mental disorders [10]. This paper reviews the research progress of histone modifications in mental disorders, discusses their mechanisms of action in various psychiatric conditions, and considers their role in understanding disease mechanisms and clinical variability. Future research directions in this field are also presented.

## 2. Association Between Histone Modifications and Mental Disorders

The main types of histone modifications include methylation, acetylation, and phosphorylation. These can alter chromatin structure and function, thereby affecting gene transcription activity [11]. Zhang *et al*., (2021) [12] provided a detailed review on the basic research and nomenclature of histones. Among these modifications, histone acetylation is one of the most extensively studied. Acetylation increases the positive charge of histones, leading to a more relaxed chromatin structure and consequently enhancing gene transcription efficiency [13]. Histone methylation is another important modification, primarily occurring on the lysine residues of histone H3 [14]. Methylation can occur as mono-, di-, or tri-methylation, and different sites and degrees of methylation exert distinct regulatory effects on gene transcription [14, 15, 16]. For instance, H3K4me3 is a hallmark of transcriptionally active promoters, facilitating the recruitment of RNA polymerase II and gene activation, whereas H3K9me2/3 and H3K27me3 are classical repressive marks that promote heterochromatin formation and transcriptional silencing. In contrast, H3K36me3 and H3K79me2 are enriched within gene bodies and contribute to transcriptional elongation and regulation of splicing. Histone phosphorylation occurs predominantly on the serine residues of histone H3 [17] and can alter the affinity between histones and DNA, thereby affecting chromatin stability.

**Fig. 1.**
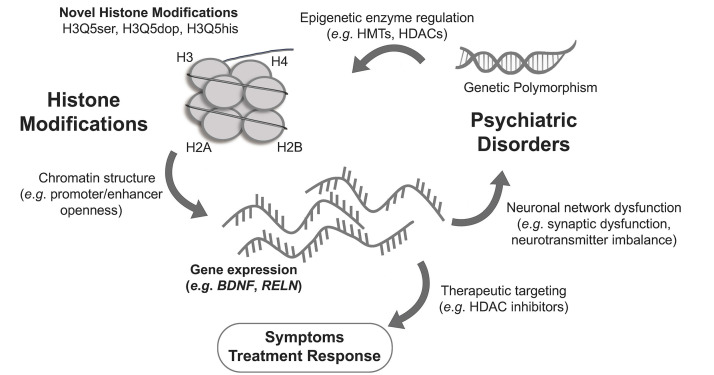
**Crosstalk among histone modifications, genetic variation, and psychiatric disorders**. This schematic illustrates the multi-layered interactions that link genetic polymorphisms, histone modifications, and psychiatric pathophysiology. Canonical and novel histone modifications such as serotonylation (H3Q5ser), dopaminylation (H3Q5dop), and histaminylation (H3Q5his) can modulate chromatin structure, accessibility of promoters/enhancers, and the transcriptional activity of neuronal genes (e.g., *BDNF*, *RELN*). Genetic variants influence the expression and activity of epigenetic enzymes (e.g., HMTs, HDACs), thereby affecting individual vulnerability to psychiatric disorders. Dysregulated gene expression contributes to neuronal network dysfunction, synaptic dysregulation, and neurotransmitter imbalance, which manifest as core symptoms of schizophrenia, bipolar disorder, and depression. Therapeutic interventions such as HDAC inhibitors act to restore chromatin balance and normalize transcriptional output, leading to improved clinical symptoms and treatment response. BDNF, brain-derived neurotrophic factor; RELN, Reelin; HMTs, histone methyltransferases; HDACs, histone deacetylases.

In psychiatric disorders, abnormal histone modifications act as key intermediates linking genetic predisposition to altered gene expression and clinical manifestations (Fig. 1). Genetic polymorphisms, such as single nucleotide polymorphisms (SNPs), can influence the expression or activity of histone-modifying enzymes, thereby modulating epigenetic regulation. These enzymes, including histone methyltransferases (HMTs) and histone deacetylases (HDACs), shape chromatin accessibility by adding or removing histone marks such as H3K27ac, H3K4me3, or H3K9me3. When chromatin remodeling disrupts neuronal gene transcription, it impairs synaptic plasticity and connectivity, driving cognitive and mood disturbances. Therapeutic agents such as HDAC inhibitors can help restore balanced chromatin states and normalize gene expression. For example, studies have shown that aberrant histone modifications in patients with schizophrenia are closely associated with pathological processes such as neurodevelopmental disorders, abnormal neuronal connectivity, and cognitive dysfunction [18]. In addition, altered levels of histone methylation in the promoter regions of certain genes have been observed in the brains of patients with depression. These changes may lead to aberrant gene expression, which subsequently affecting neuronal plasticity and function, and are associated with the emergence of depressive symptoms [19]. The role of histone modifications in various mental disorders is receiving increasing attention, and the following sections will present research progress on histone modifications in different mental disorders.

## 3. Schizophrenia

Schizophrenia is a complex neuropsychiatric disorder whose pathogenesis is influenced by both genetic and environmental factors. In recent years, epigenetic studies have revealed the important role of histone modifications in the development and progression of schizophrenia (Table 1, Ref. [20, 21, 22, 23, 24, 25]). Histone modifications play a significant role in the neurodevelopmental processes underlying schizophrenia, and abnormal histone modifications can lead to disruptions in neuronal differentiation, migration, and synapse formation, thereby affecting normal brain development and function [26, 27]. Moreover, histone modifications are linked to the dysregulation of neurotransmitter systems in the brains of schizophrenia patients. Dysfunction of the dopamine system, for example, is closely associated with changes in histone modifications [28].

**Table 1.** **Histone modifications in Schizophrenia**.

Histone Modification	Specific Site(s)	Direction of Change in Schizophrenia	Proposed Mechanism	Reported in Other Disorders?	Reference
Acetylation	Histone H3 Acetylation: H3K9ac, H3K14ac, H3K18ac	Decreased (in *Brd1* knockout mouse brain)	CNS-specific knockout of the *BRD1* gene impairs its scaffold function and associated histone acetyltransferase complex activity. *BRD1* depletion leads to increased proteolytic cleavage of the histone H3 N-terminal tail, although the upstream mechanism is unclear.	No	Paternoster *et al*., 2021 [20]
Acetylation	Histone H3 N-tail Clipping	Increased (in Brd1 knockout mouse brain)			
Acetylation	Histone H2A.Z Acetylation: combinatorial acetylation (H2A.Z.1/2K4acK7acK11ac)	Increased	Combinatorial hyperacetylation was identified on histone variants H2A.Z and H4 in patient-derived neurons. The BET family protein BRD4 is characterized as a bona fide “reader” of H2A.Zac, and its inhibition ameliorates gene expression abnormalities.	Cancer	Farrelly *et al*., 2022 [21]
Acetylation	Histone H4 Acetylation: combinatorial H4 acetylation (H4K5acK8acK16ac)	Increased			
Acetylation	Histone H3 Acetylation: H3K9ac, H3K27ac	Increased	Global reduction in histone deacetylase activity accompanied by decreased HDAC activity and decreased HDAC4 protein expression.	No	Martínez-Peula *et al*., 2024 [22]
Methylation	Histone H3 Methylation: H3K4me3	Increased			
Methylation	H3K9me2: Global (Parietal Cortex)	Increased	Forms a restrictive chromatin state, contributing to widespread transcriptional downregulation; correlated with increased HMT expression (GLP, SETDB1).	Huntington’s Disease	Chase *et al*., 2013 [23]
Methylation	H3K4me3: *ADRA2A* promoter	Increased	In the dorsolateral prefrontal cortex of individuals with schizophrenia, *ADRA2A* and *ADRA2C* gene expression is regulated by a combination of bivalent chromatin marks, the coexistence of the activating H3K4me3 and the repressive H3K27me3, together with H4K16ac. Antipsychotic treatment appears to enhance H4K16ac, thereby promoting *ADRA2A* transcription. These findings suggest an interactive regulatory mechanism linking drug exposure, epigenetic modulation, and gene-expression changes in schizophrenia.	Aging, Cancer, Autism	Brocos-Mosquera *et al*., 2021 [24]
Methylation	H3K27me3: *ADRA2A* promoter	Increased			
Methylation	H3K27me3: *ADRA2C* promoter	Increased			
Acetylation	H3K9ac: *ADRA2C* promoter	Increased			
Acetylation	H4K5ac: *ADRA2C* promoter	Increased			
Phosphorylation	H3S10	Increased	Aberrant activation of MAPK, JAK-STAT, and NF-κB signaling pathways (via cytokine, stress, or neurotransmitter input) leads to increased histone H3 serine 10 phosphorylation, associated with open chromatin and active transcription; potentially contributes to dysregulated gene expression in SCZ PBMCs.	No	Sharma *et al*., 2015 [25]

BET, bromodomain and extraterminal domain; CNS, central nervous system; HDAC, histone deacetylase; Brd1, bromodomain containing 1; HMT, histone methyltransferase; GLP, G9a-like protein; SETDB1, SET-domain, bifurcated 1; ADRA2A, adrenoceptor alpha 2A; ADRA2C, adrenoceptor alpha 2C; SCZ, schizophrenia; MAPK, mitogen-activated protein kinase; JAK-STAT, Janus kinase-signal transducer and activator of transcription; NF-κB, nuclear factor-κB; PBMCs, peripheral blood mononuclear cells.

Research into the epigenetic mechanisms of schizophrenia has revealed complex alterations in histone acetylation [20, 21, 22]. Paternoster *et al*., (2021) [20] demonstrated that central nervous system (CNS)-specific inactivation of the schizophrenia-associated gene BRD1 in mice resulted in decreased acetylation of histone H3 at lysines 9, 14, and 18 (H3K9ac, H3K14ac, H3K18ac), alongside increased histone H3 N-tail clipping. This established a crucial role for BRD1 in maintaining normal histone H3 acetylation levels in the brain, while linking its deficiency to a specific histone hypoacetylation profile. Conversely, Farrelly *et al*., (2022) [21] identified a pattern of combinatorial hyperacetylation on the histone variants H2A.Z and H4 in hiPSC-derived neurons from schizophrenia patients, a finding validated in postmortem human brain tissue. This study further characterized BRD4, a bromodomain and extraterminal domain (BET) family protein, as a direct “reader” of acetylated H2A.Z, and showed that pharmacological inhibition of BET proteins could rescue transcriptional deficits associated with the disease. Adding another layer of complexity, Martínez-Peula *et al*., (2024) [22] reported a global increase in permissive histone marks, including H3K9ac, H3K27ac, and H3K4me3, in the postmortem dorsolateral prefrontal cortex of individuals with schizophrenia. This hyperacetylation phenotype was linked to reduced HDAC activity and lower HDAC4 protein expression and was particularly pronounced in individuals with detectable levels of antipsychotic medication at the time of death. Collectively, these findings demonstrate that schizophrenia involves a dysregulated histone acetylation landscape. This includes both histone variant-specific hyperacetylation (H2A.Z/H4) and residue-specific effects on H3 that can be either hypo- or hyper-acetylated, likely depending on the genetic background, brain region, and medication status.

Emerging evidence firmly establishes aberrant histone methylation as a critical epigenetic mechanism contributing to schizophrenia and shaping gene expression programs involved in neurodevelopment, synaptic signaling, and stress response [23, 24, 29, 30]. A seminal study by Chase *et al*., (2013) [23] was the first to demonstrate a hyper-restrictive epigenomic landscape in schizophrenia. This was marked by elevated expression of the histone methyltransferases (HMTs) G9a-like protein (GLP) and SET-domain, bifurcated 1 (SETDB1), along with increased global levels of the repressive mark H3K9me2 in both postmortem parietal cortex and peripheral lymphocytes. Building on this foundation, Li *et al*., (2023) [30] uncovered a precise gene-regulatory mechanism in which SETDB1, via H3K9me3, represses the endogenous retroelement RMER21B. Loss of SETDB1 derepresses this element, enabling it to act as a distal enhancer for the *Htr3a* gene through chromatin looping. This increased enhancer activity leads to the hyperdevelopment and hyperexcitability of Htr3a-positive GABAergic interneurons, resulting in anxiety- and depression-like behaviors in mice. Such phenotypes are commonly comorbid with schizophrenia. This work provides a direct mechanistic link between HMT activity, specific neuronal subtypes, and behavioral phenotypes. Adding further nuance, Brocos-Mosquera *et al*., (2021) [24] identified promoter-specific histone methylation changes in two noradrenergic genes, *ADRA2A* and *ADRA2C*, in the dorsolateral prefrontal cortex of schizophrenia patients. Notably, the *ADRA2A* promoter exhibited a bivalent chromatin state, marked by concurrent enrichment of both H3K4me3 (activating) and H3K27me3 (repressive) modifications. This poised state may underlie the context-dependent upregulation of *ADRA2A* mRNA observed in patients exposed to antipsychotic medication, potentially facilitated by a concomitant increase in H4K16ac, which is a permissive acetylation mark. Lastly, Rahman and McGowan (2022) [29] emphasized the developmental origins of histone methylation disturbances by reviewing how early life stress (ELS) induces cell-type-specific histone modification patterns in neurons and glia. ELS-driven epigenetic reprogramming, including changes in H3K4me3 and H3K27me3, may “prime” the brain toward greater vulnerability to schizophrenia and other psychiatric conditions later in life.

Aberrantly increased histone phosphorylation in schizophrenia has been implicated in chromatin instability and dysregulated gene expression. Notably, phosphorylation of histone H3 at serine 10 (H3S10ph) is markedly elevated in patients with schizophrenia [25]. This modification is typically associated with chromatin relaxation and transcriptional activation. It may reduce histone–DNA affinity, thereby enhancing the accessibility of regulatory regions. The observed increase in H3S10ph likely reflects aberrant activation of upstream signaling pathways that are responsive to cytokine and neurotransmitter signaling, including mitogen-activated protein kinase (MAPK), Janus kinase-signal transducer and activator of transcription (JAK–STAT), and nuclear factor-κB (NF‑κB) cascades.

In addition to the classical histone modifications, emerging non-canonical modifications, such as serotonylation, lactylation, and dopaminylation, have attracted increasing attention for their potential roles in the pathogenesis of schizophrenia [18]. These neurotransmitter-derived or metabolite-driven histone marks act as integrators of neuronal signaling and chromatin regulation. We provide a dedicated section below (“Novel Histone Modifications Mediated by Neurotransmitters”) to review these findings in detail, including specific modification sites (e.g., H3Q5), functional consequences, and their relevance to psychiatric phenotypes.

Overall, histone modifications influence the pathological processes of schizophrenia through multiple mechanisms, including alteration of chromatin structure, regulation of gene transcription, and by affecting the synthesis and release of neurotransmitters. These abnormal modifications are closely related to the clinical manifestations of schizophrenia and provide potential targets for developing new therapeutic strategies. Future research will further elucidate the specific mechanisms by which histone modifications contribute to schizophrenia, offering new insights and approaches for clinical diagnosis and treatment.

## 4. Bipolar Disorder

Bipolar disorder (BD) is a chronic, disabling condition characterized by alternating mood episodes, including depressive and manic/hypomanic phases [31]. The clear influence of environmental factors, the clinical variability between manic and depressive episodes that may lead to the identification of state and trait biomarkers, and the known effects of mood stabilizers on the epigenome lay the groundwork for epigenetic research in BD [32]. Compared with schizophrenia, the studies to date on histone modifications in BD have been relatively limited and have primarily focused on the mechanisms involving histone acetylation (Table 2, Ref. [33, 34, 35]).

**Table 2.** **Histone modifications in BD**.

Histone Modification	Specific Site(s)	Direction of Change in BD	Proposed Mechanism	Reported in Other Disorders?	Reference
Acetylation	H3K9ac, H3K14ac	Decreased (at *GAD1* promoter)	Transcriptional repression of key neuronal genes (e.g., *GAD1*).	Schizophrenia	Tang *et al*., 2011 [33]
Acetylation	Genome-wide H3	Increased	Histone H3 hyperacetylation and phosphorylation may create an overly permissive chromatin environment, enhancing neuroinflammatory and stress-response gene expression, while promoter-specific hypermethylation of synaptic and plasticity-related genes (e.g., *BDNF*) reduces their transcription.	Alzheimer’s Disease	Rao *et al*., 2012 [34]
Phosphorylation	Genome-wide H3	Increased			
Chromatin organization	3D Genome Architecture	Increased/Decreased	BD may involve epigenetic remodeling of enhancer acetylation landscapes (H3K27ac) in cortical neurons, driving large-scale chromatin reorganization and transcriptional misregulation, distinct from the more genetically anchored methylation changes seen in schizophrenia.	Schizophrenia	Girdhar *et al*., 2022 [35]

BD, Bipolar disorder; BDNF, brain-derived neurotrophic factor; GAD1, glutamate decarboxylase 1.

Although evidence for altered histone acetylation in BD is starting to emerge, there is still a scarcity of locus‑level, cell type-specific ChIP‑seq data. Tang *et al*., (2011) [33] examined postmortem prefrontal cortical tissue from individuals with BD, schizophrenia (SCZ), and healthy controls, focusing on acetylation of histone H3 at lysines 9 and 14 (H3K9/K14ac). These marks are typically associated with open chromatin and active transcription. The levels of promoter-associated H3K9/K14ac in BD patients showed a significant age-dependent decline, mirroring the pattern observed in healthy individuals. In contrast, SCZ samples showed low H3K9/K14ac levels from an early age that persisted with aging, suggesting an early-onset and static hypoacetylation profile unique to SCZ. Complementing these findings, Rao *et al*., (2012) [34] reported significantly higher levels of global histone H3 acetylation levels in the frontal cortex of BD patients compared to age-matched controls. Notably, this increase was specific to BD and was not found in patients with Alzheimer’s disease (AD) [34]. The acetylation changes co-occurred with increased H3 phosphorylation (H3S10ph) and global DNA hypermethylation, suggesting coordinated epigenetic dysregulation.

A major advancement in our understanding of histone modifications in BD comes from the large-scale integrative epigenomic study by Girdhar *et al*. (2022) [35]. This postmortem study mapped nucleosomal histone acetylation landscapes in the adult prefrontal cortex of 388 controls and 351 individuals diagnosed with schizophrenia or BD [35]. The authors identified thousands of cis-regulatory domains (CRDs) and clusters of co-modified chromatin segments. They also found clusters of hyperacetylated CRDs that were shared between SCZ and BD. These regions were enriched for regulatory elements involved in fetal neurodevelopment and glutamatergic signaling, suggesting convergent disruption of neurodevelopmental transcriptional programs. Notably, SCZ showed significant enrichment of genetic risk loci within these hyperacetylated CRDs, implying that H3K27ac dysregulation in SCZ may be driven primarily by genetically encoded mechanisms that perturb chromatin architecture and gene regulation.

Valproic acid (VPA) is a widely prescribed mood stabilizer for BD that acts partially through direct epigenetic modulation [36, 37]. Seminal studies by Phiel *et al*. (2001) [36] and Göttlicher *et al*., (2001) [37] first identified VPA as a direct inhibitor of HDACs. VPA was shown to induce global hyperacetylation of histones H3 and H4, resulting in a more relaxed chromatin configuration and enhanced transcription of genes implicated in neuroplasticity, neuronal differentiation, and mood regulation. These preclinical findings were extended *in vivo* by Tseng *et al*., (2020) [38], who utilized [^11^C] Martinostat positron emission tomography (PET) to quantify HDAC expression in the living human brain. Their study revealed significantly reduced HDAC binding in the right amygdala of individuals with BD compared to healthy controls, with additional exploratory reductions observed in the bilateral thalamus, orbitofrontal cortex, and right hippocampus. Importantly, decreased HDAC expression in fronto-limbic regions was associated with emotional dysregulation and attentional impairments, two core symptom domains of BD.

These findings suggest that altered HDAC levels in BD may reflect compensatory epigenetic adaptations to chronic affective instability, cumulative medication exposure, or underlying disease mechanisms. They further highlight regional HDAC dysregulation as a promising molecular biomarker for symptom dimensions in BD.

## 5. Depression

Depression is a common psychiatric disorder with a complex pathogenesis involving the interaction of multiple biological and environmental factors [39]. Histone modifications have been shown to play an important role in the development and progression of depression [19, 40]. In particular, histone acetylation can affect neuronal plasticity and function. Altered levels of histone acetylation in certain brain regions of depressed patients may be associated with neuronal atrophy and dysfunction. Furthermore, modifications to histone methylation also play a crucial role in depression. For example, Cruceanu *et al*., (2013) [41] showed that aberrant modifications such as H3K4me3 are closely linked to dysfunctions in the neurotransmitter systems within the depressed brain.

Research on histone methylation in depression has already yielded some findings (Table 3, Ref. [30, 41, 42, 43, 44, 45, 46, 47, 48, 49, 50, 51, 52, 53, 54, 55, 56]). H3K4me3 is one of the most characteristic histone modifications and is associated with activation of gene transcription, whereas other forms such as H3K9me2 and H3K27me3 act to repress gene transcription [57]. Studies on the role of H3K4me3 in depression have mainly focused on the *Synapsin 1* and the *GDNF* genes. Cruceanu *et al*., (2013) [41] reported significant enrichment of H3K4me3 at the promoter of Synapsin II (SYN2) in postmortem brain samples from individuals with BD and major depressive disorder (MDD). This enrichment was positively correlated with increased expression of the disease-specific transcript isoforms, SYN2a in BD, and SYN2b in MDD, suggesting isoform-specific epigenetic regulation of synaptic genes via H3K4me3. In contrast, no such consistent correlation was observed at the SYN1 promoter, highlighting a potentially unique role for SYN2 regulation in affective disorders. In mice exposed to chronic unpredictable mild stress (CUMS), a reduced level of H3K4me3 in the *GDNF* gene promoter led to decreased GDNF expression, indicating that histone modifications play a key role in behavioral responses to chronic stress [42]. An increased level of H3K9me2 in the promoter region of the CaMKIIα gene has been reported to inhibit CaMKIIα expression in depressed patients and in mice treated with antidepressant [43]. Moreover, enhanced H3K27me3 in the promoter region of the *RAC1* gene in the nucleus accumbens (NAc) of mice subjected to chronic social defeat stress (CSDS) was associated with reduced RAC1 expression [44].

**Table 3.** **Histone modifications in Depression**.

Histone Modification	Specific Site(s)	Direction of Change in Depression	Proposed Mechanism	Reported in Other Disorders?	Reference
Methylation	H3K4me3: *SYN2* promoter	Increased	Increased H3K4me3 at *SYN2* promoter correlates with SYN2a (in BD) and SYN2b (in MDD) overexpression; suggests epigenetic upregulation of synapsin genes in mood disorders.	Bipolar Disorder	Cruceanu *et al*., 2013 [41]
Methylation	H3K4me3: *GDNF* promoter	Decreased (in CUMS mice)	Decreased H3K4me3 at *GDNF* promoter leads to decrease *GDNF* expression; *GDNF* is involved in neuroprotection and plasticity, linked to depressive-like behaviors in chronic stress model.	No	Uchida *et al*., 2011 [42]
Methylation	H3K9me3: RMER21B retrotransposon at *Htr3a* locus	Decreased (in Setdb1-NS-cKO mice)	Loss of SETDB1 reduces H3K9me3, derepressing the RMER21B enhancer, which loops to the *Htr3a* promoter to drive its expression and alter interneuron development.	Implicated in affective disorders	Li *et al*., 2023 [30]
Methylation	H3K9me2: CaMKIIα promoter (NAc)	Increased (in CSDS mice)	Epigenetic suppression of CaMKIIα via increased H3K9me2; fluoxetine induces repressive chromatin state that reduces CaMKIIα expression.	Schizophrenia	Robison *et al*., 2014 [43]
Methylation	H3K27me3: RAC1 promoter & upstream (NAc)	Increased (in CSDS mice)	Chronic social stress increases H3K27me3 and decreased *Rac1* expression to promotes social avoidance; reversed by HDAC inhibitor (MS-275).	No	Golden *et al*., 2013 [44]
Methylation	H3K9me3	Increased (in CK-Setdb1 mice)	Overexpression of SETDB1 in forebrain neurons induces H3K9 trimethylation at *NR2B*, promoting repressive chromatin looping and downregulating *NR2B* expression. This alters NMDA receptor subunit composition, leading to antidepressant-like behavior through reduced excitatory signaling and altered synaptic plasticity.	Bipolar affective disorder and schizophrenia	Jiang *et al*., 2010 [45]
Methylation	H3K9me2: Genome-wide (mouse NAc)	Decreased after cocaine; mimics stress vulnerability	Cocaine reduces G9a-mediated H3K9me2, enhancing BDNF-TrkB-CREB signaling and promoting susceptibility to social defeat stress.	Cocaine addiction	Covington *et al*., 2011 [46]
Methylation	H3K27me3: PFC & hippocampus	Decreased (in CSDS mice)	JMJD3 demethylates repressive H3K27me3 mark, potentially derepressing cytokine genes in response to early adolescent stress.	Mood disorders	Wang *et al*., 2018 [47]
Acetylation	H3K9/14ac, H3K18ac, H4K5/8/12/16ac	H3K9/14ac, H3K18ac, H4K5/8/12/16ac increased in DR, mPFC, vHPC for non-resilient rats; Decreased H4K8ac for both resilient and non-resilient rats	Altered histone acetylation in stress-sensitive brain regions (e.g., DR, mPFC, vHPC) correlates with behavioral susceptibility to chronic social defeat stress.	No	Kenworthy *et al*., 2014 [48]
Acetylation	H3ac, H3 phosphoacetylation, H4ac (promoters of c-fos, BDNF, CREB)	Increased H3 and H4 acetylation/phosphoacetylation after ECS; sustained H3ac at BDNF P3/P4 promoters linked to chronic upregulation	ECS induces chromatin remodeling at activity-dependent gene promoters, enhancing transcription of neuroplasticity-related genes such as BDNF and CREB, which may underlie its antidepressant efficacy.	No	Tsankova *et al*., 2004 [49]
Acetylation	H3K9ac, H4K12ac	Dynamic shift from early hypoacetylation to later hyperacetylation/methylation following repeated social defeat	SD induces time-dependent chromatin remodeling in hippocampus—initial transcriptional repression followed by activation of plasticity and reward-related genes, enhancing vulnerability to cocaine reward; Increased HAT and decreased HDAC contribute to altered chromatin accessibility.	Addiction	Montagud-Romero *et al*., 2016 [50]
Deacetylation	Hdac2 expression	Increased Hdac2 in NAc from stress-susceptible mice	No	No	Krishnan *et al*., 2007 [51]
Deacetylation	Global histone acetylation (NAc)	HDAC inhibition (MS-275, sodium butyrate); Increased histone acetylation; Decreased depressive-like behaviors	HDAC inhibitors promote gene expression via increased histone acetylation in nucleus accumbens, producing antidepressant effects.	No	Covington *et al*., 2009 [52]
Deacetylation	HDAC5 (Class II HDAC)	Decreased HDAC5 nuclear localization in stress; Increased HDAC5 nuclear accumulation after imipramine	Chronic stress reduces HDAC5 function (cytoplasmic retention), leading to stress-sensitive gene expression; imipramine restores nuclear HDAC5 to suppress maladaptive transcription.	Chronic, not acute, cocaine or stress.	Renthal *et al*., 2007 [53]
Deacetylation	*SIRT1* gene (Class III HDACs)	GWAS: SNPs in SIRT1 associated with MDD in Han Chinese females	SIRT1 regulates chromatin structure and neurotrophic signaling; implicated in both genetic risk and stress response pathways in depression.	Metabolism	CONVERGE Consortium, 2015 [54]
Deacetylation	SIRT1-BDNF signaling (PFC, Hippocampus)	Increased SIRT1 activation; Increased BDNF expression; mimics antidepressant effects	SIRT1 enhances BDNF transcription via Nrf2 signaling pathway, mediating neuroplastic and behavioral resilience to stress.	No	Yao *et al*., 2021 [55]
Crotonylation	H3 crotonylation (e.g., H3K9cr, H3K18cr in mPFC)	Decreased in medial prefrontal cortex of stress-susceptible mice	Upregulation of CDYL reduces histone crotonylation and increases H3K27me3 at the *VGF* promoter, repressing transcription and impairing synaptic plasticity and mood regulation.	No	Liu *et al*., 2019 [56]

DR, dorsal raphe; GWAS, genome-wide association studies; MDD, major depressive disorder; CDYL, chromodomain Y-like protein; CUMS, chronic unpredictable mild stress; CSDS, chronic social defeat stress; NMDA, N-methyl-D-aspartate; TrkB, tropomyosin receptor kinase B; PFC, prefrontal cortex; *NR2B*, *N-methyl-D-aspartate receptor 2B subunit*; CSDS, chronic social defeat stress; ECS, electroconvulsive stimulation; SNP, single nucleotide polymorphisms; VGF, nerve growth factor inducible; CREB, cAMP-response element-binding protein; Nrf2, *nuclear factor erythroid 2-related factor 2*; HDAC5, histone deacetylase 5; NAc, nucleus accumbens.

Histone methyltransferases (HMTs) also play important roles in depression. SETDB1, a histone methyltransferase, has been implicated in H3K9me3-mediated gene repression and has emerged as a key modulator of mood-related behavior. Jiang *et al*., (2010) [45] demonstrated that enhanced Setdb1 expression in forebrain neurons produced antidepressant-like effects in mice, including reduced behavioral despair in the tail suspension and forced swim tests. More recently, SETDB1 was found to regulate the development of Htr3a-positive interneurons in the cortex, which are essential for proper emotion regulation. Furthermore, Setdb1 haploinsufficiency led to enhanced depression- and anxiety-like phenotypes in both male and female mice [30]. G9a (EHMT2) is a lysine methyltransferase that catalyzes the repressive epigenetic mark H3K9me2 and has been shown to play a central role in mood regulation. In a seminal study, Covington *et al*., (2011) [46] demonstrated that chronic cocaine exposure followed by social defeat stress (SDS) in mice led to decreased G9a expression and reduced H3K9me2 levels in NAc, a brain region central to reward and affect regulation. Liu *et al*., (2019) [58] demonstrated that genetic deletion of PRMT1, a key protein arginine methyltransferase, ameliorated depressive-like behaviors in mice. This behavioral resilience was accompanied by upregulated expression of brain-derived neurotrophic factor (BDNF) and postsynaptic density protein 95 (PSD95) in the hippocampus and prefrontal cortex, indicating that PRMT1-mediated histone arginine methylation may repress synaptic plasticity–related gene expression under stress conditions [58]. In a complementary study, Wang *et al*., (2018) [47] reported that CUMS exposure in adolescent rats led to upregulation of the H3K27me3-specific demethylase JMJD3 in both the prefrontal cortex and hippocampus, resulting in global reduction of the repressive H3K27me3 mark.

Histone acetylation is also critically involved in depression. Studies by Tsankova *et al*. [49, 59] demonstrated that histone acetylation was associated with the depressive state in a mouse model of depression induced by CSDS. Treatment with the antidepressant imipramine effectively induced histone acetylation to alleviate depression. Specifically, expression of the BDNF gene was suppressed in the hippocampus of depressed mice. However, imipramine treatment increased H3 acetylation in the BDNF promoter region, leading to upregulated BDNF expression. Moreover, RAC1 gene expression decreased following CSDS, which was linked to reduced H3 acetylation in its promoter region. Research by Kenworthy *et al*. [48] and Montagud-Romero *et al*. [50] found reduced levels of H3K9ac, H3K14ac, H4K5ac, H4K8ac, H4K12ac, and H4K16ac in the dorsal raphe (DR) and hippocampus of mice following SDS, whereas the acetylation levels of H4K12 and H3K14 increased 15 or 21 days after the stress exposure.

The role of HDACs in depression has also been extensively studied. Human HDACs are classified into four classes: Class I (HDAC1, 2, 3, 8), Class II (HDAC4, 5, 6, 7, 9, 10), Class III (SIRTs), and Class IV (HDAC11). Among them, Class I and Class II HDACs are the most critical in regulating depression [19]. The NAc is a core brain region involved in reward and mood. Stress susceptibility was associated with increased HDAC2 expression and decreased histone acetylation following SDS, while antidepressant treatment reduced HDAC2 levels and restored acetylation of histone H3 at lysines 14 and 27 [51, 52]. Histone deacetylase 5 (HDAC5), a Class II member, was shown to accumulate in the nucleus upon chronic imipramine treatment, leading to the suppression of stress-responsive genes and promotion of resilience. Conversely, HDAC5-deficient mice exhibit increased sensitivity to chronic emotional stress and impaired behavioral adaptation [53]. Beyond central expression, genome-wide association studies (GWAS) have identified SNPs within the *SIRT1* gene, a Class III HDAC, as significantly associated with MDD in Han Chinese females, implicating SIRT1-mediated histone and metabolic regulation in the risk of depression [54]. Experimental studies further revealed that activation of SIRT1 enhances BDNF expression and mediates the antidepressant effects of nuclear factor erythroid 2-related factor 2 (Nrf2) agonists, suggesting an epigenetic-metabolic bridge in MDD pathophysiology [55]. HDAC1 has also emerged as a stress-responsive factor in peripheral systems. Although primarily studied in regard to endothelial function, HDAC1 is recognized as a potential environmental sensor that may influence stress-induced behavioral phenotypes through vascular or immune-related mechanisms [60]. These findings indicate that both histone acetylation and HDACs play significant roles in the pathogenesis of depression, offering potential targets for the development of new therapeutic strategies.

Recent evidence has identified chromodomain Y-like protein (CDYL) as a key regulator of histone crotonylation in stress-induced depression [56]. Liu *et al*., (2019) [56] reported that chronic social defeat stress leads to increased CDYL expression and concomitant reductions in histone crotonylation levels in the medial prefrontal cortex of depressed mice. Although these changes correlate with depressive-like behaviors, causality remains to be fully established. Nevertheless, mechanistic experiments indicate that CDYL can repress the transcription of neuropeptide genes such as nerve growth factor inducible (*VGF*) by reducing histone crotonylation and increasing H3K27me3 at their promoters, thereby linking environmental stress to epigenetic modulation of mood-related pathways.

In summary, research into the mechanisms of histone modification in psychiatric disorders is providing new perspectives for understanding the molecular pathology of these conditions and for exploring effective, mechanism-based therapeutic targets.

## 6. Emerging Modifications and Technologies

### 6.1 Novel Histone Modifications Mediated by Neurotransmitters

Neurotransmitter-mediated histone modifications are a recently discovered and groundbreaking category of histone post-translational modifications. These directly link brain signaling molecules to chromatin regulation, thus providing a new framework for understanding how neuronal activity and environmental cues shape gene expression. As mentioned above, one of the earliest and most studied forms is histone serotonylation [61, 62, 63], in whereby serotonin is covalently attached to the glutamine 5 residue on histone H3 (H3Q5ser) [64] by transglutaminase 2 (TGM2) [65]. This modification often co-occurs with H3K4me3, a marker of active promoters, and has been shown to facilitate transcription initiation in serotonergic neurons [66]. Similarly, dopaminylation, the covalent addition of dopamine to H3Q5 (H3Q5dop), has been identified in dopaminergic neurons of the ventral tegmental area [67], as well as in non-dopaminergic regions such as the NAc [68]. This modification plays a key role in regulating gene expression programs involved in reward processing, addiction, and behavioral plasticity, particularly in response to cocaine exposure [67, 69]. Importantly, the dysregulation of dopamine-linked histone marks such as H3Q5dop may extend beyond addiction to psychiatric disorders that are characterized by dopaminergic imbalance. For example, aberrant dopaminergic signaling in the ventral tegmental area (VTA)-NAc circuit is a well-established feature of anhedonia and motivational deficits in depression. Another neurotransmitter-derived modification is histaminylation (H3Q5his), which is found in the hypothalamus and is implicated in circadian rhythms [70]. These modifications demonstrate that neurotransmitters function not only as synaptic messengers but also as epigenetic modifiers, bridging extracellular signals with chromatin-based transcriptional regulation and offering novel insights into the neuroepigenetic landscape.

### 6.2 Advances in Single-Cell and Spatial Epigenomics

The development of single-cell epigenomic technologies has revolutionized our understanding of histone modifications in heterogeneous brain tissues. Tools such as: scCUT&Tag (single-cell cleavage under targets and tagmentation) [71] and scChIP-seq [72] allow the profiling of histone modifications with single-cell resolution, revealing cell type-specific chromatin landscapes in both healthy and diseased brains [73]. Furthermore, spatial epigenomics platforms that combine chromatin profiling with tissue imaging, enable the localization of histone modifications within intact brain structures [74]. Emerging computational frameworks can now integrate histone modification maps with transcriptomics, chromatin conformation (Hi-C), DNA methylation, and even proteomics to identify regulatory circuits underlying mental illness. Machine learning methods, including SHAP-enhanced feature selection and deep chromatin state prediction [75], are increasingly applied to prioritize functional epigenetic biomarkers and therapeutic targets.

Together, these advances underscore a new paradigm in neuroepigenetics, where neurotransmitters not only modulate behavior but also write epigenetic memory into the genome. By leveraging innovative technologies, researchers are now poised to elucidate in detail how specific histone modifications contribute to disease risk, symptom heterogeneity, and treatment response at unprecedented resolution [76].

### 6.3 Histone Modifications as Therapeutic Targets for Mental Disorders

The critical role of histone modifications in mental disorders has made them a focal point in the development of therapeutic strategies [10]. Among these, HDACs and modifiers of histone methylation have emerged as key molecular targets. HDAC inhibitors (HDACi) exert antidepressant and anxiolytic effects primarily by increasing histone acetylation to enhance chromatin accessibility and promote the expression of neuroplasticity-related genes [77, 78]. Mechanistically, HDACi neutralize the positive charges on histone tails, thus weakening histone-DNA interactions and relaxing chromatin structure. This facilitates the transcriptional activation of genes that are critical for neuronal function. For example, studies have shown that HDACi can increase the expression of BDNF [79], a key molecule involved in neuronal survival, differentiation, and plasticity [80]. The resulting upregulation of BDNF leads to the alleviation of depressive and anxiety symptoms.

Therapeutic approaches that target modifications to histone methylation are also being explored. Abnormal methylation can be rectified by modulating the activity of HMTs or demethylases. For instance, some studies have found that adjusting histone H3K4 methylation can affect gene expression. H3K4 methylation is generally associated with gene activation, whereas H3K9 methylation is linked to gene repression [81, 82]. By modulating these methylation marks, aberrant gene expression can be corrected, thereby improving the symptoms of mental disorders.

Future research should aim to explore in further detail the specific mechanisms by which histone modifications contribute to various mental disorders, and how targeting such modulations could lead to precise treatment approaches. This should ultimately lead to more effective therapeutic strategies, thus improving patients’ quality of life.

## 7. Challenges and Future Perspectives

The mechanisms by which histone modifications influence mental disorders are complex and multifaceted, involving neural development, neurotransmitter regulation, and neuronal plasticity [8]. With ongoing research advances, histone modifications hold promise not only as diagnostic markers but also as therapeutic targets for mental disorders.

However, despite promising evidence implicating the role of histone modifications in the pathophysiology of mental disorders, the field continues to face critical methodological and interpretative challenges that limit the robustness and generalizability of current findings.

One prominent example is the inconsistent directionality of H3K4me3 changes across studies and disorders. H3K4me3 is a classical transcriptionally permissive mark that has been reported to increase in the prefrontal cortex of individuals with MDD in some postmortem studies, consistent with upregulation of stress-responsive genes. However, other studies using a rodent CSDS model observed region-specific reductions in H3K4me3, particularly in the hippocampus and NAc. These were associated with behavioral despair and social avoidance phenotypes. Results are similarly mixed with regard to schizophrenia. Some human postmortem analyses reported widespread reductions of H3K4me3 in promoter regions of synaptic and glutamatergic genes, whereas others found increased H3K4me3 occupancy at pro-inflammatory gene loci. The divergent findings suggest that several factors may strongly modulate the pattern and consequences of H3K4me3 alterations, including cell-type specificity, brain region, developmental stage, and disease subtype. Moreover, dynamic H3K4me3 changes may occur during different phases of disease progression or treatment response, which is rarely considered in cross-sectional designs. These contradictory findings highlight the need for precise, high-resolution approaches—such as ChIP-seq in sorted cell populations, single-nucleus epigenomics, and CUT&Tag methods—to resolve whether H3K4me3 alterations are disease drivers, consequences, or compensatory mechanisms.

Second, many studies rely on postmortem human brain tissue, which is subject to confounding factors such as cause of death, agonal state, medication history, and postmortem interval, all of which can alter the histone marks. Moreover, cell-type heterogeneity in bulk tissue analyses can obscure neuron- or glia-specific epigenetic dynamics. While some recent work incorporates single-cell or cell-sorted epigenomics, such approaches remain technically demanding and limited in scale.

Third, significant translational gaps exist between rodent models and human depression. Rodent models (e.g., chronic social defeat stress, learned helplessness) capture only select facets of depressive phenotypes and do not fully recapitulate the heterogeneity observed in human patients. Additionally, sex-specific and developmental-stage-specific epigenetic regulation is often overlooked, despite growing evidence of their relevance in determining vulnerability to psychiatric disease.

Finally, the relative strength of evidence varies widely across histone marks and disorders. For example, histone acetylation and HDAC inhibition are supported by robust preclinical and pharmacological data in depression and anxiety, whereas the roles of newer modifications such as crotonylation or serotonylation remain preliminary and largely correlative.

Future studies should further elucidate the specific roles of histone modifications in different mental disorders. They should also explore how the modulation of histone modifications can lead to more precise treatments. Such efforts will be crucial in developing more effective therapeutic strategies and improving the overall clinical outcomes and quality of life for patients suffering from these conditions.

## References

[1] Ruthenburg AJ, Li H, Patel DJ, Allis CD (2007). Multivalent engagement of chromatin modifications by linked binding modules. *Nature Reviews. Molecular Cell Biology*.

[2] Liu M, Yue Y, Chen X, Xian K, Dong C, Shi M (2025). Genome-coverage single-cell histone modifications for embryo lineage tracing. *Nature*.

[3] Xu Y, Zhao W, Olson SD, Prabhakara KS, Zhou X (2018). Alternative splicing links histone modifications to stem cell fate decision. *Genome Biology*.

[4] Regadas I, Dahlberg O, Vaid R, Ho O, Belikov S, Dixit G (2021). A unique histone 3 lysine 14 chromatin signature underlies tissue-specific gene regulation. *Molecular Cell*.

[5] Geng H, Chen H, Wang H, Wang L (2021). The Histone Modifications of Neuronal Plasticity. *Neural Plasticity*.

[6] Day JJ, Sweatt JD (2011). Epigenetic mechanisms in cognition. *Neuron*.

[7] Zhang M, Zhao J, Lv Y, Wang W, Feng C, Zou W (2020). Histone Variants and Histone Modifications in Neurogenesis. *Trends in Cell Biology*.

[8] Park J, Lee K, Kim K, Yi SJ (2022). The role of histone modifications: from neurodevelopment to neurodiseases. *Signal Transduction and Targeted Therapy*.

[9] Bannister AJ, Kouzarides T (2011). Regulation of chromatin by histone modifications. *Cell Research*.

[10] Marques D, Vaziri N, Greenway SC, Bousman C (2025). DNA methylation and histone modifications associated with antipsychotic treatment: a systematic review. *Molecular Psychiatry*.

[11] Millán-Zambrano G, Burton A, Bannister AJ, Schneider R (2022). Histone post-translational modifications - cause and consequence of genome function. *Nature Reviews*.

[12] Zhang Y, Sun Z, Jia J, Du T, Zhang N, Tang Y, Fang D, Han J (2021). Overview of Histone Modification. *Histone Mutations and Cancer*.

[13] Chakravarty S, Bhat UA, Reddy RG, Gupta P, Kumar A, Peedicayil J, Grayson DR, Avramopoulos D (2021). Histone Deacetylase Inhibitors and Psychiatric Disorders. *Epigenetics in Psychiatry*.

[14] Peter CJ, Akbarian S (2011). Balancing histone methylation activities in psychiatric disorders. *Trends in Molecular Medicine*.

[15] Greer EL, Shi Y (2012). Histone methylation: a dynamic mark in health, disease and inheritance. *Nature Reviews. Genetics*.

[16] Shi YG, Tsukada Y (2013). The discovery of histone demethylases. *Cold Spring Harbor Perspectives in Biology*.

[17] Li J, Mahata B, Escobar M, Goell J, Wang K, Khemka P (2021). Programmable human histone phosphorylation and gene activation using a CRISPR/Cas9-based chromatin kinase. *Nature Communications*.

[18] Chen YZ, Zhu XM, Lv P, Hou XK, Pan Y, Li A (2024). Association of histone modification with the development of schizophrenia. *Biomedicine & Pharmacotherapy*.

[19] Wu MS, Li XJ, Liu CY, Xu Q, Huang JQ, Gu S (2022). Effects of Histone Modification in Major Depressive Disorder. *Current Neuropharmacology*.

[20] Paternoster V, Edhager AV, Qvist P, Donskov JG, Shliaha P, Jensen ON (2021). Inactivation of the Schizophrenia-associated BRD1 gene in Brain Causes Failure-to-thrive, Seizure Susceptibility and Abnormal Histone H3 Acetylation and N-tail Clipping. *Molecular Neurobiology*.

[21] Farrelly LA, Zheng S, Schrode N, Topol A, Bhanu NV, Bastle RM (2022). Chromatin profiling in human neurons reveals aberrant roles for histone acetylation and BET family proteins in schizophrenia. *Nature Communications*.

[22] Martínez-Peula O, Morentin B, Callado LF, Meana JJ, Rivero G, Ramos-Miguel A (2024). Permissive epigenetic regulatory mechanisms at the histone level are enhanced in postmortem dorsolateral prefrontal cortex of individuals with schizophrenia. *Journal of Psychiatry & Neuroscience: JPN*.

[23] Chase KA, Gavin DP, Guidotti A, Sharma RP (2013). Histone methylation at H3K9: evidence for a restrictive epigenome in schizophrenia. *Schizophrenia Research*.

[24] Brocos-Mosquera I, Miranda-Azpiazu P, Muguruza C, Corzo-Monje V, Morentin B, Meana JJ (2021). Differential brain ADRA2A and ADRA2C gene expression and epigenetic regulation in schizophrenia. Effect of antipsychotic drug treatment. *Translational Psychiatry*.

[25] Sharma RP, Feiner B, Chase KA (2015). Histone H3 phosphorylation is upregulated in PBMCs of schizophrenia patients in comparison to healthy controls. *Schizophrenia Research*.

[26] Girdhar K, Hoffman GE, Jiang Y, Brown L, Kundakovic M, Hauberg ME (2018). Cell-specific histone modification maps in the human frontal lobe link schizophrenia risk to the neuronal epigenome. *Nature Neuroscience*.

[27] Feiner B, Chase KA, Melbourne JK, Rosen C, Sharma RP (2019). Risperidone effects on heterochromatin: the role of kinase signaling. *Clinical and Experimental Immunology*.

[28] Abdolmaleky HM, Zhou JR, Thiagalingam S (2021). Cataloging recent advances in epigenetic alterations in major mental disorders and autism. *Epigenomics*.

[29] Rahman MF, McGowan PO (2022). Cell-type-specific epigenetic effects of early life stress on the brain. *Translational Psychiatry*.

[30] Li J, Zheng S, Dong Y, Xu H, Zhu Y, Weng J (2023). Histone Methyltransferase SETDB1 Regulates the Development of Cortical Htr3a-Positive Interneurons and Mood Behaviors. *Biological Psychiatry*.

[31] Ludwig B, Dwivedi Y (2016). Dissecting bipolar disorder complexity through epigenomic approach. *Molecular Psychiatry*.

[32] Legrand A, Iftimovici A, Khayachi A, Chaumette B (2021). Epigenetics in bipolar disorder: a critical review of the literature. *Psychiatric Genetics*.

[33] Tang B, Dean B, Thomas EA (2011). Disease- and age-related changes in histone acetylation at gene promoters in psychiatric disorders. *Translational Psychiatry*.

[34] Rao JS, Keleshian VL, Klein S, Rapoport SI (2012). Epigenetic modifications in frontal cortex from Alzheimer’s disease and bipolar disorder patients. *Translational Psychiatry*.

[35] Girdhar K, Hoffman GE, Bendl J, Rahman S, Dong P, Liao W (2022). Chromatin domain alterations linked to 3D genome organization in a large cohort of schizophrenia and bipolar disorder brains. *Nature Neuroscience*.

[36] Phiel CJ, Zhang F, Huang EY, Guenther MG, Lazar MA, Klein PS (2001). Histone deacetylase is a direct target of valproic acid, a potent anticonvulsant, mood stabilizer, and teratogen. *The Journal of Biological Chemistry*.

[37] Göttlicher M, Minucci S, Zhu P, Krämer OH, Schimpf A, Giavara S (2001). Valproic acid defines a novel class of HDAC inhibitors inducing differentiation of transformed cells. *The EMBO Journal*.

[38] Tseng CEJ, Gilbert TM, Catanese MC, Hightower BG, Peters AT, Parmar AJ (2020). In vivo human brain expression of histone deacetylases in bipolar disorder. *Translational Psychiatry*.

[39] Flint J (2023). The genetic basis of major depressive disorder. *Molecular Psychiatry*.

[40] Cheng J, Hu H, Ju Y, Liu J, Wang M, Liu B (2024). Gut microbiota-derived short-chain fatty acids and depression: deep insight into biological mechanisms and potential applications. *General Psychiatry*.

[41] Cruceanu C, Alda M, Nagy C, Freemantle E, Rouleau GA, Turecki G (2013). H3K4 tri-methylation in synapsin genes leads to different expression patterns in bipolar disorder and major depression. *The International Journal of Neuropsychopharmacology*.

[42] Uchida S, Hara K, Kobayashi A, Otsuki K, Yamagata H, Hobara T (2011). Epigenetic status of GDNF in the ventral striatum determines susceptibility and adaptation to daily stressful events. *Neuron*.

[43] Robison AJ, Vialou V, Sun HS, Labonte B, Golden SA, Dias C (2014). Fluoxetine epigenetically alters the CaMKIIα promoter in nucleus accumbens to regulate ΔFosB binding and antidepressant effects. *Neuropsychopharmacology: Official Publication of the American College of Neuropsychopharmacology*.

[44] Golden SA, Christoffel DJ, Heshmati M, Hodes GE, Magida J, Davis K (2013). Epigenetic regulation of RAC1 induces synaptic remodeling in stress disorders and depression. *Nature Medicine*.

[45] Jiang Y, Jakovcevski M, Bharadwaj R, Connor C, Schroeder FA, Lin CL (2010). Setdb1 histone methyltransferase regulates mood-related behaviors and expression of the NMDA receptor subunit NR2B. *The Journal of Neuroscience: the Official Journal of the Society for Neuroscience*.

[46] Covington HE, Maze I, Sun H, Bomze HM, DeMaio KD, Wu EY (2011). A role for repressive histone methylation in cocaine-induced vulnerability to stress. *Neuron*.

[47] Wang R, Wang W, Xu J, Liu D, Jiang H, Pan F (2018). Dynamic Effects of Early Adolescent Stress on Depressive-Like Behaviors and Expression of Cytokines and JMJD3 in the Prefrontal Cortex and Hippocampus of Rats. *Frontiers in Psychiatry*.

[48] Kenworthy CA, Sengupta A, Luz SM, Ver Hoeve ES, Meda K, Bhatnagar S (2014). Social defeat induces changes in histone acetylation and expression of histone modifying enzymes in the ventral hippocampus, prefrontal cortex, and dorsal raphe nucleus. *Neuroscience*.

[49] Tsankova NM, Kumar A, Nestler EJ (2004). Histone modifications at gene promoter regions in rat hippocampus after acute and chronic electroconvulsive seizures. *The Journal of Neuroscience: the Official Journal of the Society for Neuroscience*.

[50] Montagud-Romero S, Montesinos J, Pascual M, Aguilar MA, Roger-Sanchez C, Guerri C (2016). ‘Up-regulation of histone acetylation induced by social defeat mediates the conditioned rewarding effects of cocaine. *Progress in Neuro-psychopharmacology & Biological Psychiatry*.

[51] Krishnan V, Han MH, Graham DL, Berton O, Renthal W, Russo SJ (2007). Molecular adaptations underlying susceptibility and resistance to social defeat in brain reward regions. *Cell*.

[52] Covington HE, Maze I, LaPlant QC, Vialou VF, Ohnishi YN, Berton O (2009). Antidepressant actions of histone deacetylase inhibitors. *The Journal of Neuroscience: the Official Journal of the Society for Neuroscience*.

[53] Renthal W, Maze I, Krishnan V, Covington HE, Xiao G, Kumar A (2007). Histone deacetylase 5 epigenetically controls behavioral adaptations to chronic emotional stimuli. *Neuron*.

[54] CONVERGE consortium (2015). Sparse whole-genome sequencing identifies two loci for major depressive disorder. *Nature*.

[55] Yao W, Lin S, Su J, Cao Q, Chen Y, Chen J (2021). Activation of BDNF by transcription factor Nrf2 contributes to antidepressant-like actions in rodents. *Translational Psychiatry*.

[56] Liu Y, Li M, Fan M, Song Y, Yu H, Zhi X (2019). Chromodomain Y-like Protein-Mediated Histone Crotonylation Regulates Stress-Induced Depressive Behaviors. *Biological Psychiatry*.

[57] Yuan M, Yang B, Rothschild G, Mann JJ, Sanford LD, Tang X (2023). Epigenetic regulation in major depression and other stress-related disorders: molecular mechanisms, clinical relevance and therapeutic potential. *Signal Transduction and Targeted Therapy*.

[58] Liu H, Jiang J, Zhao L (2019). Protein arginine methyltransferase-1 deficiency restrains depression-like behavior of mice by inhibiting inflammation and oxidative stress via Nrf-2. *Biochemical and Biophysical Research Communications*.

[59] Tsankova NM, Berton O, Renthal W, Kumar A, Neve RL, Nestler EJ (2006). Sustained hippocampal chromatin regulation in a mouse model of depression and antidepressant action. *Nature Neuroscience*.

[60] Dunaway LS, Pollock JS (2022). HDAC1: an environmental sensor regulating endothelial function. *Cardiovascular Research*.

[61] Fu L, Zhang L (2019). Serotonylation: A novel histone H3 marker. *Signal Transduction and Targeted Therapy*.

[62] Zlotorynski E (2019). Histone serotonylation boosts neuronal transcription. *Nature Reviews. Molecular Cell Biology*.

[63] Al-Kachak A, Di Salvo G, Fulton SL, Chan JC, Farrelly LA, Lepack AE (2024). Histone serotonylation in dorsal raphe nucleus contributes to stress- and antidepressant-mediated gene expression and behavior. *Nature Communications*.

[64] Zhao S, Chuh KN, Zhang B, Dul BE, Thompson RE, Farrelly LA (2021). Histone H3Q5 serotonylation stabilizes H3K4 methylation and potentiates its readout. *Proceedings of the National Academy of Sciences of the United States of America*.

[65] Lukasak BJ, Mitchener MM, Kong L, Dul BE, Lazarus CD, Ramakrishnan A (2022). TGM2-mediated histone transglutamination is dictated by steric accessibility. *Proceedings of the National Academy of Sciences of the United States of America*.

[66] Farrelly LA, Thompson RE, Zhao S, Lepack AE, Lyu Y, Bhanu NV (2019). Histone serotonylation is a permissive modification that enhances TFIID binding to H3K4me3. *Nature*.

[67] Lepack AE, Werner CT, Stewart AF, Fulton SL, Zhong P, Farrelly LA (2020). Dopaminylation of histone H3 in ventral tegmental area regulates cocaine seeking. *Science (New York, N.Y.)*.

[68] Stewart AF, Lepack AE, Fulton SL, Safovich P, Maze I (2023). Histone H3 dopaminylation in nucleus accumbens, but not medial prefrontal cortex, contributes to cocaine-seeking following prolonged abstinence. *Molecular and Cellular Neurosciences*.

[69] Fulton SL, Mitra S, Lepack AE, Martin JA, Stewart AF, Converse J (2022). Histone H3 dopaminylation in ventral tegmental area underlies heroin-induced transcriptional and behavioral plasticity in male rats. *Neuropsychopharmacology: Official Publication of the American College of Neuropsychopharmacology*.

[70] Zheng Q, Weekley BH, Vinson DA, Zhao S, Bastle RM, Thompson RE (2025). Bidirectional histone monoaminylation dynamics regulate neural rhythmicity. *Nature*.

[71] Wu SJ, Furlan SN, Mihalas AB, Kaya-Okur HS, Feroze AH, Emerson SN (2021). Single-cell CUT&Tag analysis of chromatin modifications in differentiation and tumor progression. *Nature Biotechnology*.

[72] Liu Z, Deng C, Zhou Z, Xiao Y, Jiang S, Zhu B (2024). Epigenomic tomography for probing spatially defined chromatin state in the brain. *Cell Reports Methods*.

[73] Zenk F, Fleck JS, Jansen SMJ, Kashanian B, Eisinger B, Santel M (2024). Single-cell epigenomic reconstruction of developmental trajectories from pluripotency in human neural organoid systems. *Nature Neuroscience*.

[74] Lu T, Ang CE, Zhuang X (2022). Spatially resolved epigenomic profiling of single cells in complex tissues. *Cell*.

[75] Wang Y, Kong S, Zhou C, Wang Y, Zhang Y, Fang Y (2024). A review of deep learning models for the prediction of chromatin interactions with DNA and epigenomic profiles. *Briefings in Bioinformatics*.

[76] Yao W, Hu X, Wang X (2024). Crossing epigenetic frontiers: the intersection of novel histone modifications and diseases. *Signal Transduction and Targeted Therapy*.

[77] Volmar C-H, Wahlestedt C (2015). Histone deacetylases (HDACs) and brain function. *Neuroepigenetics*.

[78] Liu T, Wan Y, Xiao Y, Xia C, Duan G (2020). Dual-Target Inhibitors Based on HDACs: Novel Antitumor Agents for Cancer Therapy. *Journal of Medicinal Chemistry*.

[79] Seo MK, Kim YH, McIntyre RS, Mansur RB, Lee Y, Carmona NE (2018). Effects of Antipsychotic Drugs on the Epigenetic Modification of Brain-Derived Neurotrophic Factor Gene Expression in the Hippocampi of Chronic Restraint Stress Rats. *Neural Plasticity*.

[80] Varela-Andrés N, Cebrián-León A, Hernández-del Caño C, Fernández del Campo IS, García-Losada S, Martín-Ávila N (2024). MSK1 MSK1 absence hinders BDNF-dependent striatal neurodevelopment and leads to schizophrenia symptoms. *bioRxiv*.

[81] Okazaki S, Boku S, Otsuka I, Horai T, Kimura A, Shimmyo N (2021). Clozapine increases macrophage migration inhibitory factor (MIF) expression via increasing histone acetylation of MIF promoter in astrocytes. *Journal of Psychiatric Research*.

[82] Su Y, Liu X, Lian J, Deng C (2020). Epigenetic histone modulations of PPARγ and related pathways contribute to olanzapine-induced metabolic disorders. *Pharmacological Research*.

